# Risk Factors Associated with Uncomplicated Peptic Ulcer and Changes in Medication Use after Diagnosis

**DOI:** 10.1371/journal.pone.0101768

**Published:** 2014-07-08

**Authors:** Antonio González-Pérez, María E. Sáez, Saga Johansson, Péter Nagy, Luis A. García Rodríguez

**Affiliations:** 1 The Spanish Centre for Pharmacoepidemiologic Research (CEIFE), Madrid, Spain; 2 Andalusian Bioinformatics Research Centre (CAEBi), Seville, Spain; 3 AstraZeneca R&D, Mölndal, Sweden; Charité, Campus Benjamin Franklin, Germany

## Abstract

**Background:**

Few epidemiologic studies have investigated predictors of uncomplicated peptic ulcer disease (PUD) separately from predictors of complicated PUD.

**Objective:**

To analyze risk factors associated with uncomplicated PUD and medication use after diagnosis.

**Methods:**

Patients diagnosed with uncomplicated PUD (n = 3,914) were identified from The Health Improvement Network database among individuals aged 40–84 years during 1997–2005, with no previous history of PUD. Prescription records for the year after the date of diagnosis were reviewed and a nested case–control analysis was performed to calculate the odds ratios for the association of potential risk factors with PUD.

**Results:**

Medications associated with developing uncomplicated PUD included current use of acetylsalicylic acid (ASA), nonsteroidal anti-inflammatory drugs (NSAIDs), paracetamol, selective serotonin reuptake inhibitors, antidepressants, antihypertensives or acid suppressants. Uncomplicated PUD was significantly associated with being a current or former smoker and having had a score of at least 3 on the Townsend deprivation index. Approximately 50% of patients who were users of ASA (19% of patients) or chronic users of NSAIDs (7% of patients) at diagnosis did not receive another prescription of the medication in the 60 days after diagnosis, and 30% were not represcribed therapy within a year. Among patients who were current users of ASA or chronic NSAIDs at the time of the PUD diagnosis and received a subsequent prescription for their ASA or NSAID during the following year, the vast majority (80–90%) also received a proton pump inhibitor coprescription.

**Conclusions:**

Our results indicate that several risk factors for upper gastrointestinal bleeding are also predictors of uncomplicated PUD, and that some patients do not restart therapy with ASA or NSAIDs after a diagnosis of uncomplicated PUD. Further investigation is needed regarding the consequences for these patients in terms of increased cardiovascular burden due to discontinuation of antiplatelet therapy.

## Introduction

In the UK general population, it has been estimated that the incidence of peptic ulcer complications, including ulcer haemorrhage or perforation, is approximately 1 per 1000 person-years, and about 5–10% of these complications may be fatal [1]–[3]. Although the need for efficient identification and treatment of potentially life-threatening complications is clear, uncomplicated peptic ulcer disease (PUD) is also clinically relevant and contributes to the overall health burden of PUD. Complications may develop in patients with initially uncomplicated ulcer [4], [5] and, even in the absence of overt bleeding, uncomplicated peptic ulcers may lead to the development of anemia [6]. Upper gastrointestinal (GI) symptoms potentially related to PUD affect patients’ health-related quality of life [7] and such symptoms have also been reported to affect patients’ use of acetylsalicylic acid (ASA) [8]. A recent observational study suggested that a history of uncomplicated PUD approximately doubles the probability of poor adherence to nonsteroidal anti-inflammatory drug (NSAID) therapy [9].

We have previously shown that, from 1997 to 2005, the overall incidence of uncomplicated PUD was 0.75 cases per 1000 person-years in a study conducted using The Health Improvement Network (THIN), a large, UK-based primary care database [10]. Incidences of uncomplicated PUD of a similar magnitude were reported in a recent population-based study in Denmark [4]. Observational data probably reflect the incidence of symptomatic uncomplicated ulcer, given that asymptomatic ulcers are likely to remain undiagnosed. While risk factors for PUD overall and upper GI complications in particular have been well studied [1], [2], [11]–[15], few studies have investigated risk factors associated specifically with symptomatic uncomplicated PUD. Such information could aid the early identification of patients who would benefit from monitoring or treatment.

In the present analysis, we have built on our previous observational study of symptomatic uncomplicated PUD [10]. We performed a nested case–control analysis using the same population from THIN [10] to identify predictors of uncomplicated PUD in the general population, with a focus on the association with medication use. We also investigated changes in prescribing of medications after diagnosis of uncomplicated PUD.

## Materials and Methods

### Data Source

Data were collected from THIN, a computerized primary care database containing anonymized records for over 3 million individuals currently registered with participating primary care practices in the UK. Patients included in the database are representative of the general UK population with respect to age, sex and geographical region [16]. Information contained in THIN includes patient demographics, details of consultations with primary care physicians (PCPs), information about consultant referrals and hospitalizations, laboratory test results, diagnoses and prescriptions. Diagnoses and symptoms are recorded using Read codes [17]. The validity of THIN for use in pharmacoepidemiologic studies has been demonstrated [18].

### Study Population

Selection of the study population has been described in detail elsewhere [10]. Briefly, patients were identified who were aged 40–84 years between January 1997 and December 2005, who had been enrolled with their PCP for at least 2 years and who had at least 1 year of computerized prescription history. The date when a patient met the inclusion criteria was that individual’s start date. Patients were excluded if they had received a diagnosis of cancer, Mallory–Weiss syndrome or PUD (complicated or uncomplicated), or if they had a history of upper GI bleeding, esophageal varices, alcohol abuse, liver disease or coagulopathies. Patients were also excluded if they were aged 70 years or over at their start date and had at least 1 year of follow-up with one or fewer data points recorded during that time (proxy for incomplete data recording). All remaining patients constituted the study population (N = 1,049,689).

### Uncomplicated PUD Case Ascertainment and Control Population Selection

All patients in the study population were followed up until the end of the study period (December 2005) or until they reached the age of 85 years, died, met any of the exclusion criteria (including receiving a diagnosis of complicated PUD) or received a diagnosis of uncomplicated PUD. Of the individuals who had a Read code suggestive of a diagnosis of uncomplicated PUD, true cases (n = 3,914) were considered to be those for whom the diagnosis had been made during a specialist visit or hospitalization (typically involving endoscopy – the standard diagnostic technique in the UK) [10], [19]. A random sample of 143 cases of uncomplicated PUD was used previously to demonstrate the positive predictive rate: 94% were confirmed as definite or possible cases [10]. In addition, 9,969 control individuals with no diagnosis of uncomplicated PUD were sampled from the whole study cohort using incidence density sampling, which is the method of choice for obtaining unbiased results in a nested case–control analysis [20]. Briefly, a random date was selected for each individual in the study cohort who did not receive a diagnosis of uncomplicated PUD; if this date fell within the individual’s eligible person-time (i.e. before they met an exclusion criterion), this individual was considered an eligible control. Thus, the likelihood of being selected as a control was proportional to the individual’s person-time at risk. Controls were frequency-matched to cases by age, sex and year of start date.

It was observed that many (n = 1,764) of the patients who received a diagnosis of uncomplicated PUD had recorded upper GI symptoms (including nausea, vomiting, dyspepsia, heartburn and epigastric pain) before their date of PUD diagnosis. The mean length of time between early symptom appearance and diagnosis of uncomplicated PUD for all patients with such early symptoms was found to be 131 days. Based on these data, for all patients with uncomplicated PUD, the index date used for nested case–control analysis was 120 days (4 months) before the recorded date of diagnosis of uncomplicated PUD. For controls, the index date for nested case–control analysis was the randomly chosen date generated during the control selection process.

### Data Collection and Analysis

In the present analysis (Clinicaltrials.gov: NCT01888588), for all cases and controls, demographic, lifestyle and comorbidity information was ascertained at the index date, as well as information on exposure to medications. Medication use was classified as current, recent, past and non-use as follows: current use, prescription active at the index date or ending in the 7 days before the index date; recent use, most recent prescription ending 8–30 days before the index date; past use, most recent prescription ending 31–365 days before the index date; and non-use, no recorded prescription or most recent prescription ending more than 365 days before the index date. The group of current ASA users was subdivided for particular analyses: naïve current ASA users were defined as those receiving ASA at the index date and having received their first ASA prescription during the study period after an interval of more than 365 days during which they received no ASA prescription. All other ASA users were defined as non-naïve.

Social deprivation was measured in all patients according to the Townsend deprivation index [21]. We were able to ascertain *Helicobacter pylori* infection status among patients with uncomplicated PUD, as described previously [10], but not among control individuals. Therefore, comparisons were made with the overall control group to estimate the risk of *H. pylori* positive and *H. pylori* negative uncomplicated PUD associated with use of different drugs. Nested case–control analyses were carried out using unconditional logistic regression analysis models, adjusted (when appropriate) for sex, age, year of index date, number of PCP visits and specialist referrals in the year before the index date, smoking status and use of gastroprotective drugs (proton pump inhibitors [PPIs] or histamine type 2 receptor antagonists [H_2_RAs]), paracetamol, ASA and NSAIDs. All statistical analyses were carried out using Stata SE (version 12.0; StataCorp, College Station, Texas, USA).

For patients who received an uncomplicated PUD diagnosis, ASA, NSAID and PPI use was also ascertained in the year following their date of diagnosis (as recorded in their medical records).

The analysis of data from the THIN database was approved by the Multicentre Research Ethics Committee (REC reference 07/MRE05/18) and patient records were anonymized and de-identified prior to analysis. All authors had access to the study data and reviewed and approved the final manuscript.

## Results

### Patient Characteristics and Comorbidities

The demographics and lifestyle characteristics at the index date for the 3,914 patients who received a diagnosis of uncomplicated PUD during the study period and the 9,969 controls are shown in Table 1, along with comorbidities significantly associated with the development of uncomplicated PUD (the unadjusted odds ratios [OR] are presented in Table S1). Uncomplicated PUD was significantly associated with being a current or former smoker, having had at least two PCP visits or one or more specialist referrals in the year before the index date, and having had a score of at least 3 on the Townsend deprivation index. Among the comorbidities, stress, depression, gastroesophageal reflux disease and having upper GI symptoms (including nausea, vomiting, dyspepsia, heartburn and epigastric pain) were all significantly associated with uncomplicated PUD development.

**Table 1:** Patient demographics and lifestyle characteristics at the index date, and comorbidities significantly associated with uncomplicated PUD development, in a UK primary care population during 1997–2005.

	Cases	Controls	Association^a^
	n = 3,914	n = 9,969	
	n (%)	n (%)	OR (95% CI)
Sex			
Male	1,921 (49.1)	4,893 (49.1)	NA
Female	1,993 (50.9)	5,076 (50.9)	NA
Age, years			
40–49	669 (17.1)	1,712 (17.2)	NA
50–59	896 (22.9)	2,276 (22.8)	NA
60–69	1,023 (26.1)	2,619 (26.3)	NA
70–79	1,030 (26.3)	2,601 (26.1)	NA
80–84	296 (7.5)	761 (7.6)	NA
Smoking status			
Non-smoker	1,725 (44.1)	5,292 (53.1)	1.00
Smoker	1,151 (29.4)	1,852 (18.6)	1.90 (1.72–2.10)
Former smoker	680 (17.4)	1,431 (14.4)	1.30 (1.15–1.45)
Unknown	358 (9.1)	1,394 (14.0)	0.97 (0.85–1.11)
Alcohol use^b^			
None/occasional	2,046 (52.3)	4,619 (46.3)	1.00
Light	856 (21.9)	2,430 (24.4)	0.86 (0.78–0.95)
Moderate	199 (5.1)	473 (4.7)	0.97 (0.80–1.17)
Heavy	133 (3.4)	332 (3.3)	0.86 (0.69–1.08)
Unknown	680 (17.4)	2,115 (21.2)	1.00 (0.87–1.15)
BMI, kg/m^2^			
<20	161 (4.1)	337 (3.4)	1.16 (0.94–1.44)
20–24	1,066 (27.2)	2,744 (27.5)	1.00
25–29	1,258 (32.1)	3,047 (30.6)	1.00 (0.90–1.11)
≥30	695 (17.8)	1,605 (16.1)	0.93 (0.82–1.05)
Unknown	734 (18.8)	2,236 (22.4)	1.04 (0.91–1.19)
PCP visits in the previous year			
0–1	1,417 (36.2)	4,735 (47.5)	1.00
2–5	1,463 (37.4)	3,537 (35.5)	1.17 (1.06–1.29)
≥6	1,034 (26.4)	1,697 (17.0)	1.48 (1.31–1.67)
Specialist referrals in the previous year			
0	2,685 (68.6)	7,822 (78.5)	1.00
1–2	804 (20.5)	1,537 (15.4)	1.26 (1.13–1.40)
≥3	425 (10.9)	610 (6.1)	1.34 (1.16–1.56)
Hospitalization in the previous year			
0	3,688 (94.2)	9,657 (96.9)	1.00
≥1	226 (5.8)	312 (3.1)	1.21 (0.99–1.48)
Townsend deprivation index			
0 (least deprived)	231 (5.9)	603 (6.0)	1.10 (0.91–1.32)
1	779 (19.9)	2,546 (25.5)	1.00
2	792 (20.2)	2,227 (22.3)	1.08 (0.96–1.22)
3	782 (20.0)	1,926 (19.3)	1.16 (1.03–1.31)
4	741 (18.9)	1,573 (15.8)	1.27 (1.12–1.45)
5 (most deprived)	589 (15.0)	1,094 (11.0)	1.35 (1.18–1.56)
Comorbidities^c^			
Stress	246 (6.3)	430 (4.3)	1.23 (1.03–1.47)
Depression	900 (23.0)	1,615 (16.2)	1.23 (1.11–1.36)
GERD	716 (18.3)	971 (9.7)	1.19 (1.06–1.34)
Upper GI symptoms^d^	2,050 (52.4)	2,845 (28.5)	1.88 (1.72–2.04)

Abbreviations: ASA, acetylsalicylic acid; BMI, body mass index; CI, confidence interval; GERD, gastroesophageal reflux disease; GI, gastrointestinal; H_2_RA, histamine type 2 receptor antagonist; NA, not applicable (controls were frequency-matched to cases by sex and age); NSAID, nonsteroidal anti-inflammatory drug; OR, odds ratio; PCP, primary care physician; PPI, proton pump inhibitor; PUD, peptic ulcer disease.

a Relative to the indicated category, and adjusted when appropriate for sex, age, year of index date, number of PCP visits and specialist referrals in the year before the index date, smoking status, and use of gastroprotective drugs (PPIs or H_2_RAs), paracetamol, ASA and NSAIDs.

b None/occasional, <3 units per week; light, 3–15 units per week; moderate, 16–24 units per week; heavy, ≥25 units per week).

c Relative to being free from the respective comorbidity. Only significantly associated comorbidities are listed.

d Including nausea, vomiting, dyspepsia, heartburn and epigastric pain.

### Medication Use and the Risk of Uncomplicated PUD Development

Current use of ASA, NSAIDs, paracetamol, selective serotonin reuptake inhibitors, tricyclic antidepressants, antihypertensives or gastroprotective medications (PPIs and H_2_RAs) was associated with uncomplicated PUD development, relative to non-use (Table 2, the unadjusted values are presented in Table S2). For current use of ASA, the risk of uncomplicated PUD was increased 1.5-fold (OR: 1.54; 95% confidence interval [CI]: 1.37–1.74), while dual antiplatelet therapy (ASA and clopidogrel) was associated with an approximately 2.5-fold increased risk (OR: 2.62; 95% CI: 1.12–6.11).

**Table 2:** Medications for which current, past or recent use was significantly associated with uncomplicated PUD development in a UK primary care population during 1997–2005.

	Cases	Controls	Association^a^
	n = 3,914	n = 9,969	
	n (%)	n (%)	OR (95% CI)
ASA			
Current	725 (18.5)	1,133 (11.4)	1.54 (1.37–1.74)
Recent	65 (1.7)	89 (0.9)	1.74 (1.23–2.46)
Past	142 (3.6)	233 (2.3)	1.28 (1.01–1.61)
NSAIDs			
Current	560 (14.3)	734 (7.4)	1.70 (1.49–1.94)
Recent	103 (2.6)	212 (2.1)	1.04 (0.80–1.34)
Past	560 (14.3)	1,303 (13.1)	1.03 (0.91–1.16)
Oral anticoagulants			
Current	85 (2.2)	154 (1.5)	1.21 (0.91–1.61)
Recent	14 (0.4)	27 (0.3)	1.31 (0.66–2.62)
Past	32 (0.8)	34 (0.3)	1.81 (1.09–3.02)
Paracetamol			
Current	649 (16.6)	868 (8.7)	1.56 (1.38–1.78)
Recent	190 (4.9)	281 (2.8)	1.45 (1.18–1.78)
Past	651 (16.6)	1,140 (11.4)	1.32 (1.17–1.49)
PPIs			
Current	451 (11.5)	524 (5.3)	2.05 (1.77–2.36)
Recent	79 (2.0)	53 (0.5)	4.21 (2.92–6.08)
Past	334 (8.5)	278 (2.8)	2.89 (2.42–3.44)
H_2_RAs			
Current	261 (6.7)	215 (2.2)	2.99 (2.47–3.63)
Recent	67 (1.7)	34 (0.3)	4.62 (2.99–7.12)
Past	344 (8.8)	237 (2.4)	3.25 (2.71–3.89)
SSRIs			
Current	159 (4.1)	216 (2.2)	1.37 (1.09–1.72)
Recent	20 (0.5)	52 (0.5)	0.80 (0.46–1.38)
Past	108 (2.8)	212 (2.1)	0.92 (0.72–1.19)
Tricyclic antidepressants			
Current	205 (5.2)	279 (2.8)	1.29 (1.05–1.57)
Recent	15 (0.4)	34 (0.3)	0.77 (0.39–1.49)
Past	145 (3.7)	214 (2.1)	1.28 (1.01–1.61)
Antihypertensives			
Current	1,494 (38.2)	2,938 (29.5)	1.13 (1.02–1.26)
Recent	68 (1.7)	186 (1.9)	0.82 (0.60–1.11)
Past	165 (4.2)	310 (3.1)	1.27 (1.03–1.57)

Abbreviations: ASA, acetylsalicylic acid; CI, confidence interval; H_2_RA, histamine type 2 receptor antagonist; NSAID, nonsteroidal anti-inflammatory drug; OR, odds ratio; PCP, primary care physician; PPI, proton pump inhibitor; PUD, peptic ulcer disease; SSRI, selective serotonin reuptake inhibitor.

a Relative to non-use. Adjusted (when appropriate) according to sex, age, year of index date, number of PCP visits and specialist referrals in the year before the index date, smoking status, and use of gastroprotective drugs (PPIs or H_2_RAs), paracetamol, ASA and NSAIDs.

#### Dose, Duration of Treatment, and Number of Medications

For NSAIDs, the association was stronger in current users of multiple NSAIDs (OR: 3.82; 95% CI: 2.16–6.73) than in current users of only a single NSAID (OR: 1.64; 95% CI: 1.43–1.87), and increased with increasing duration of treatment (OR: 2.46; 95% CI: 1.92–3.16 for use of >3 years, versus OR: 1.07; 95% CI: 0.84–1.35 for use of <3 months). Current users of ASA<100 mg had a similar risk to those who received ASA 150–300 mg (<100 mg OR: 1.52; 95% CI: 1.33–1.73; 100–200 mg OR: 1.61; 95% CI: 1.27–2.04; and 200–300 mg OR: 1.70; 95% CI: 1.12–2.57). The risk of developing uncomplicated PUD was elevated whether patients had received ASA for less than 3 months or longer than 3 years (OR: 1.82; 95% CI: 1.38–2.41; and OR: 1.53; 95% CI: 1.26–1.86, respectively). ORs for the risk of developing uncomplicated PUD were 1.50 (95% CI: 1.29–1.75) and 2.41 (95% CI: 1.25–4.63) in naïve and non-naïve users of ASA, respectively. An association was also apparent between uncomplicated PUD development and current use of oral corticosteroids with duration of longer than 3 years (OR: 1.92; 95% CI: 1.04–3.53).

#### Gastric and Duodenal Ulcers

The association between medication use and uncomplicated PUD development was also analyzed according to ulcer location (Table 3, the unadjusted values are presented in Table S3). Current use of ASA was associated with an increased risk of uncomplicated gastric ulcer (OR: 1.69; 95% CI: 1.45–1.96) and of uncomplicated duodenal ulcer (OR: 1.36; 95% CI: 1.16–1.61). Current use of NSAIDs was more strongly associated with the development of uncomplicated gastric ulcer than with uncomplicated duodenal ulcer, and the results indicated that oral anticoagulant use may be associated with uncomplicated gastric ulcer, but not uncomplicated duodenal ulcer.

**Table 3:** Medications for which current, past or recent use was significantly associated with uncomplicated PUD development when stratified by ulcer location (gastric or duodenal), in a UK primary care population during 1997–2005.

	Uncomplicated gastric ulcer			Uncomplicated duodenal ulcer		
	Cases^a^	Controls	Association^b^	Cases^a^	Controls	Association^b^
	n = 1,688	n = 9,969		n = 1,721	n = 9,969	
	n (%)	n (%)	OR (95% CI)	n (%)	n (%)	OR (95%) CI
ASA						
Current	376 (22.3)	1,133 (11.4)	1.69 (1.45–1.96)	274 (15.9)	1,133 (11.4)	1.36 (1.16–1.61)
Recent	35 (2.1)	89 (0.9)	2.13 (1.39–3.25)	24 (1.4)	89 (0.9)	1.46 (0.90–2.37)
Past	69 (4.1)	233 (2.3)	1.34 (0.99–1.79)	53 (3.1)	233 (2.3)	1.11 (0.80–1.54)
NSAIDs						
Current	309 (18.3)	734 (7.4)	1.98 (1.68–2.33)	185 (10.7)	734 (7.4)	1.36 (1.13–1.64)
Recent	51 (3.0)	212 (2.1)	1.10 (0.79–1.53)	35 (2.0)	212 (2.1)	0.82 (0.56–1.21)
Past	251 (14.9)	1,303 (13.1)	1.07 (0.91–1.26)	245 (14.2)	1,303 (13.1)	1.00 (0.85–1.18)
Oral anticoagulants						
Current	47 (2.8)	154 (1.5)	1.44 (1.01–2.06)	29 (1.7)	154 (1.5)	1.02 (0.66–1.55)
Recent	9 (0.5)	27 (0.3)	2.31 (1.04–5.10)	4 (0.2)	27 (0.3)	0.70 (0.23–2.16)
Past	20 (1.2)	34 (0.3)	2.44 (1.36–4.40)	7 (0.4)	34 (0.3)	0.99 (0.42–2.29)
PPIs						
Current	207 (12.3)	524 (5.3)	1.84 (1.53–2.22)	180 (10.5)	524 (5.3)	2.03 (1.68–2.47)
Recent	33 (2.0)	53 (0.5)	3.52 (2.21–5.61)	35 (2.0)	53 (0.5)	4.56 (2.89–7.21)
Past	145 (8.6)	278 (2.8)	2.65 (2.12–3.31)	145 (8.4)	278 (2.8)	2.97 (2.37–3.73)
H_2_RAs						
Current	118 (7.0)	215 (2.2)	2.77 (2.17–3.54)	120 (7.0)	215 (2.2)	3.44 (2.70–4.37)
Recent	29 (1.7)	34 (0.3)	3.90 (2.30–6.64)	31 (1.8)	34 (0.3)	5.43 (3.26–9.05)
Past	133 (7.9)	237 (2.4)	2.59 (2.05–3.28)	177 (10.3)	237 (2.4)	3.83 (3.09–4.75)

Abbreviations: ASA, acetylsalicylic acid; CI, confidence interval; H_2_RA, histamine type 2 receptor antagonist; NSAID, nonsteroidal anti-inflammatory drug; OR, odds ratio; PCP, primary care physician; PPI, proton pump inhibitor; PUD, peptic ulcer disease.

a Data are presented for all cases for which ulcer location (gastric or duodenal) was recorded.

b Relative to non-use. Adjusted (when appropriate) according to sex, age, year of index date, number of PCP visits and specialist referrals in the year before the index date, smoking status, and use of gastroprotective drugs (PPIs or H_2_RAs), paracetamol, ASA and NSAIDs.

#### Helicobacter Pylori Infection Status

Among cases of uncomplicated PUD, 1,333 patients were determined to be positive for *H. pylori*, 368 patients were determined to be negative for *H. pylori*, and infection status was unknown in 2,213 patients. Medication use significantly associated with an increased risk of developing uncomplicated PUD in patients positive or negative for *H. pylori* is presented in Table S4. The results should be interpreted with caution, given that it is not possible to ascertain the rationale for whether or not *H. pylori* status was determined among patients in the database, and infection status was not available for control individuals.

#### Gastroprotective Medication

It was reasoned that the positive association between uncomplicated PUD development and the use of PPIs and H_2_RAs was probably due to confounding by indication (i.e. the fact that these medications are used to treat upper GI symptoms and complications). To test this hypothesis, we analyzed the association between PPI use and the risk of uncomplicated PUD development in the subgroup of naïve users of ASA. Naïve current users of ASA were split into three groups: those who did not receive a PPI prescription at any point between their first ASA prescription and their index date (non-users); those who were prescribed a PPI at the same time as their first ASA prescription; and those who were not prescribed a PPI at the time of first ASA prescription but received a PPI prescription afterwards.

Patients who received a PPI prescription sometime after their first ASA prescription had a significantly increased risk of developing uncomplicated PUD compared with non-users of a PPI (OR: 2.29; 95% CI: 1.45–3.63). In contrast, this association was not apparent among patients who received a PPI at the same time as their first ASA prescription, compared with non-users of a PPI (OR: 0.86; 95% CI: 0.42–1.78 for patients with continuous PPI use until the index date) (Table 4, the unadjusted values are presented in Table S5). These results suggest that PPI use does not increase the risk of uncomplicated PUD and that the observed association with uncomplicated PUD is due to PPI prescription to treat upper GI symptoms, possibly associated with undiagnosed PUD.

**Table 4:** Association between PPI use and uncomplicated PUD development in naïve current ASA users in a UK primary care population during 1997–2005.

	Cases	Controls	Association^a^
	n = 350	n = 541	
	n (%)	n (%)	OR (95% CI)
No PPI	253 (72.3)	452 (83.5)	1.00
PPI at first ASA prescription	38 (10.9)	49 (9.1)	1.27 (0.79–2.04)
Continuous until index date	13 (3.7)	24 (4.4)	0.86 (0.42–1.78)
Non-continuous	25 (7.1)	25 (4.6)	1.66 (0.91–3.04)
PPI added after first ASA prescription	59 (16.9)	40 (7.4)	2.29 (1.45–3.63)

Abbreviations: ASA, acetylsalicylic acid; CI, confidence interval; NSAID, nonsteroidal anti-inflammatory drug; OR, odds ratio; PCP, primary care physician; PPI, proton pump inhibitor; PUD, peptic ulcer disease.

a Relative to non-use of a PPI, and adjusted according to sex, age, year of index date, number of PCP visits and specialist referrals in the year before the index date, smoking status, and use of paracetamol and NSAIDs.

### Use of ASA and NSAIDs after Diagnosis of Uncomplicated PUD

In total, 52.9% of patients who were current users of ASA at diagnosis of uncomplicated PUD received at least one ASA prescription in the 60 days after their date of diagnosis, and 42.7% received at least two ASA prescriptions in this time period (proxy for continuous use). By 1 year after the date of diagnosis, 70.1% and 61.4% of current users of ASA at diagnosis had received at least one or at least two ASA prescriptions, respectively. Most of these patients received a PPI coprescription with ASA (Table 5). In contrast, only 19.3% (141/730) of current users of ASA at diagnosis were also receiving a PPI at their date of diagnosis. Of current chronic NSAID users (duration of treatment ≥1 year) at their date of diagnosis, 49.6% received at least one NSAID prescription in the 60 days after diagnosis, and 84.7% of these also received at least one PPI prescription. At their date of diagnosis, 20.3% (56/276) of current users of an NSAID were also receiving a PPI.

**Table 5:** ASA and NSAID prescription after diagnosis of uncomplicated PUD in current users at the date of diagnosis in a UK primary care population during 1997–2005.

Cases (n = 3,914)	60 days after date of diagnosis	180 days after date of diagnosis	365 days after date of diagnosis
	n (%)	n (%)	n (%)
Current ASA use at date of diagnosis (n = 730)			
Received at least one ASA prescription after diagnosis	386 (52.9)	493 (67.5)	512 (70.1)
Also received at least one PPI prescription after diagnosis	320/386 (82.9)	433/493 (87.8)	463/512 (90.4)
Received at least two ASA prescriptions after diagnosis	312 (42.7)	422 (57.8)	448 (61.4)
Also received at least two PPI prescriptions after diagnosis	221/312 (70.8)	304/422 (72.0)	321/448 (71.7)
Current NSAID use at date of diagnosis, chronic use^a^ (n = 276)			
Received at least one NSAID prescription after diagnosis	137 (49.6)	163 (59.1)	177 (64.1)
Also received at least one PPI prescription after diagnosis	116/137 (84.7)	147/163 (90.2)	164/177 (92.7)
Received at least two NSAID prescriptions after diagnosis	106 (38.4)	135 (48.9)	147 (53.3)
Also received at least two PPI prescriptions after diagnosis	78/106 (73.6)	102/135 (75.6)	112/147 (76.2)
Current NSAID use at date of diagnosis, non-chronic use^b^ (n = 240)			
Received at least one NSAID prescription after diagnosis	51 (21.3)	70 (29.2)	95 (39.6)
Also received at least one PPI prescription after diagnosis	37/51 (72.5)	58/70 (82.9)	82/95 (86.3)
Received at least two NSAID prescriptions after diagnosis	38 (15.8)	59 (24.6)	71 (29.6)
Also received at least two PPI prescriptions after diagnosis	26/38 (68.4)	43/59 (72.9)	51/71 (71.8)

Abbreviations: ASA, acetylsalicylic acid; NSAID, nonsteroidal anti-inflammatory drug; PPI, proton pump inhibitor; PUD, peptic ulcer disease.

a Duration ≥1 year.

b Duration <1 year.

## Discussion

Few epidemiologic studies have investigated predictors of uncomplicated PUD separately from predictors of complicated PUD. Identification of patients at risk of uncomplicated PUD is clinically relevant, given that initially uncomplicated PUD may develop into complicated PUD [4], [5], and upper GI symptoms potentially associated with uncomplicated PUD may affect patients’ health-related quality of life and adherence to therapy with ASA and NSAIDs [7]–[9]. Our results indicate that development of uncomplicated PUD is associated with the use of ASA, dual antiplatelet therapy, NSAIDs, paracetamol, PPIs, H_2_RAs, selective serotonin reuptake inhibitors, tricyclic antidepressants, antihypertensives and long-term use of oral corticosteroids; and with smoking, frequent use of healthcare services, social deprivation, stress, depression and upper GI symptoms. We also found that about 30% of patients who were current users of ASA or chronic users of NSAIDs when diagnosed with uncomplicated PUD did not receive another prescription of their medication in the year after diagnosis.

Many of the factors that were observed in the present study to increase the risk of uncomplicated PUD have also been demonstrated to increase the risk of complications of PUD. Previous studies have reported an increased risk of upper GI bleeding associated with smoking [3], [22]–[24], use of ASA or NSAIDs [13], [14], [22], [23], [25], [26], use of high doses of oral corticosteroids [22] and with use of selective serotonin reuptake inhibitors [25], [27].

Our results confirm evidence from previous observational studies that reported an increased risk of uncomplicated PUD associated with ASA and NSAID use [4], [28], [29]. The observations that we report also indicate that combinations of medications may further increase the risk of uncomplicated PUD. Compared with non-use, current use of dual antiplatelet therapy (ASA and clopidogrel) was associated with an approximately 2.5-fold increased risk of uncomplicated PUD, and use of multiple NSAIDs was associated with an almost 4-fold increased risk, although it should be noted that these estimates are based on low patient numbers. Higher doses of NSAIDs were associated with a higher risk of uncomplicated PUD than lower doses. However, no dose response was observed for ASA when comparing doses of <100 mg daily to doses of 200–300 mg daily, indicating that the elevated risk of uncomplicated PUD associated with ASA use is present across the dose range prescribed for cardioprotection (75–300 mg) and may not be reduced by lowering the dose of ASA.

GI symptoms in individuals receiving ASA or NSAID treatment may be more closely monitored than in non-users of these drugs, and this might increase the likelihood of ASA or NSAID users eventually being diagnosed with uncomplicated PUD compared with non-users (i.e. detection bias). At the same time, initial symptoms of uncomplicated PUD might lead to early discontinuation of ASA or NSAIDs followed by investigations that ultimately result in diagnosis of uncomplicated PUD (i.e. inverse protopathic bias). We attempted to minimize the effect of these biases by adjusting for health services utilization and GI symptoms in the year before the event, and by using a date earlier than that of the recorded diagnosis as the index date in the case–control analysis. However, it is difficult to predict how these two seemingly opposing biases might have affected our results. Similarly, the positive associations that we observed between uncomplicated PUD development and PPI or H_2_RA use may be due to confounding by indication. Thus, the associations with gastroprotective medications, GI symptoms and use of healthcare services are likely to be part of the natural history of uncomplicated PUD, or comorbidities, rather than being factors that directly increase the risk of PUD.

A further analysis supported the assertion that the association with gastroprotective medications was due to confounding. When the use of PPIs was analyzed specifically in naïve users of ASA, the association between PPIs and uncomplicated PUD development was observed only in individuals who received a PPI after their first ASA prescription, not in those who received a PPI at the same time as their first ASA prescription. In patients who received a PPI after their first ASA prescription, the PPI may have been prescribed to treat GI symptoms that had arisen as a result of ASA use.

Our analysis of represcribing of medications after development of uncomplicated PUD showed that about 70% of patients who were current users of ASA at diagnosis received another prescription for ASA in the year after diagnosis. Similar levels of represcribing were observed among patients who were current chronic NSAID users at diagnosis. A previous study has shown that represcription rates for ASA and NSAIDs in the year after upper GI bleeding in Denmark were 43% and 25%, respectively; most patients (approximately 88%) in that study who were represcribed ASA or an NSAID also received a PPI [30]. Similarly, in our analysis, most patients (80–90%) who received ASA or NSAID therapy after diagnosis of uncomplicated PUD received concomitant PPI therapy, in line with evidence-based guidelines [31]. However, only about 20% of current users of ASA or NSAIDs were receiving PPI therapy when diagnosed with uncomplicated PUD, indicating that patients receiving ASA or NSAIDs with other GI risk factors may not be identified and prescribed PPI therapy until uncomplicated PUD or upper GI complications are diagnosed. The patients (approximately 30%) who are current users at diagnosis but do not continue with ASA therapy may include some individuals who require continuous ASA therapy for cardioprotection, leaving them at an increased risk of cardiovascular events [32].

The present study has the strength of using a large primary care database containing a substantial amount of anonymized patient data that is representative of the UK general population, and that has been validated for use in epidemiologic studies [16], [18]. It should be noted that no information is recorded about the use of over-the-counter medications. However, prescriptions are free in the UK for patients aged over 60 years, reducing the likelihood of long-term over-the-counter medication use. Misclassification of NSAID or ASA users as non-users due to medications being obtained over the counter would tend to lead to an underestimation of the risk of uncomplicated PUD associated with ASA or NSAID use.

Taken together, the results presented here indicate that use of NSAIDs, ASA (across the cardioprotective dose range) and several other medications are risk factors for uncomplicated PUD. These and other identified risk factors, including smoking, are also risk factors for PUD complications. About 70% of patients who are users of ASA or chronic users of NSAIDs at diagnosis are represcribed their medication within a year of diagnosis of uncomplicated PUD; most of these patients also receive PPI therapy, in accordance with guidelines [31]. However, the 30% of patients who do not receive another prescription of ASA may include some individuals in need of continuous ASA therapy, who will be at increased risk of cardiovascular events compared with those who continue to receive ASA [32]. For patients with risk factors for uncomplicated PUD, it is important to take preventative measures to reduce the likelihood of ulcer development.

## Supporting Information

Table S1 **Patient demographics and lifestyle characteristics at the index date, and comorbidities significantly associated with uncomplicated PUD development.**
(DOC)

Table S2 **Medications for which current, past or recent use was significantly associated with uncomplicated PUD development.**
(DOC)

Table S3 **Medications for which current, past or recent use was significantly associated with uncomplicated PUD development when stratified by ulcer location (gastric or duodenal).**
(DOC)

Table S4 **Medication use significantly associated with uncomplicated PUD development, stratified by** ***Helicobacter pylori*** **infection status, in a UK primary care population during 1997–2005.**
(DOCX)

Table S5 **Association between PPI use and uncomplicated PUD development in naïve current ASA users.**
(DOC)
