# Supplementary material for: Risk Factors Associated with Uncomplicated Peptic Ulcer and Changes in Medication Use after Diagnosis
Source: PLoS One. 2014 Jul 8;9(7):e101768. doi: 10.1371/journal.pone.0101768 (PMC4086954; doi:10.1371/journal.pone.0101768)
Supplement: Table S1 — Patient demographics and lifestyle characteristics at the index date, and comorbidities significantly associated with uncomplicated PUD development. (DOC) [file pone.0101768.s001.doc]

***Table S1. Patient demographics and lifestyle characteristics at the index date, and comorbidities significantly associated with uncomplicated PUD development.***

|  | **Adjusted OR^a^ (95% CI)** | **Crude OR^a^ (95% CI)** |
| --- | --- | --- |
| Smoking status |  |  |
| Non-smoker | 1.00 | 1.00 |
| Smoker | 1.90 (1.72–2.10) | 1.91 (1.74–2.09) |
| Former smoker | 1.30 (1.15–1.45) | 1.46 (1.31–1.62) |
| Unknown | 0.97 (0.85–1.11) | 0.79 (0.69–0.90) |
| Alcohol use^b^ |  |  |
| None/occasional | 1.00 | 1.00 |
| Light | 0.86 (0.78–0.95) | 0.80 (0.72–0.87) |
| Moderate | 0.97 (0.80–1.17) | 0.95 (0.80–1.13) |
| Heavy | 0.86 (0.69–1.08) | 0.90 (0.73–1.11) |
| Unknown | 1.00 (0.87–1.15) | 0.73 (0.66–0.80) |
| BMI, kg/m^2^ |  |  |
| < 20 | 1.00 | 1.00 |
| 20–24 | 1.16 (0.94–1.44) | 1.23 (1.01–1.50) |
| 25–29 | 1.00 (0.90–1.11) | 1.06 (0.96–1.17) |
| ≥ 30 | 0.93 (0.82–1.05) | 1.11 (0.99–1.25) |
| Unknown | 1.04 (0.91–1.19) | 0.84 (0.7–60.94) |
| PCP visits in the previous year |  |  |
| 0–1 | 1.00 | 1.00 |
| 2–5 | 1.17 (1.06–1.29) | 1.38 (1.27–1.50) |
| ≥ 6 | 1.48 (1.31–1.67) | 2.04 (1.85–2.24) |
| Specialist referrals in the previous year |  |  |
| 0 | 1.00 | 1.00 |
| 1–2 | 1.26 (1.13–1.40) | 1.52 (1.38–1.68) |
| ≥ 3 | 1.34 (1.16–1.56) | 2.03 (1.78–2.31) |
| Hospitalization in the previous year |  |  |
| 0 | 1.00 | 1.00 |
| ≥ 1 | 1.21 (0.99–1.48) | 1.90 (1.59-2.26) |
| Townsend deprivation index |  |  |
| 0 (least deprived) | 1.10 (0.91–1.32) | 1.25 (1.05–1.49) |
| 1 | 1.00 | 1.00 |
| 2 | 1.08 (0.96–1.22) | 1.16 (1.04–1.30) |
| 3 | 1.16 (1.03–1.31) | 1.33 (1.18–1.49) |
| 4 | 1.27 (1.12–1.45) | 1.54 (1.37–1.73) |
| 5 (most deprived) | 1.35 (1.18–1.56) | 1.76 (1.55–2.00) |
| Comorbidities^c^ |  |  |
| Stress | 1.23 (1.03–1.47) | 1.49 (1.27–1.75) |
| Depression | 1.23 (1.11–1.36) | 1.54 (1.41–1.69) |
| GERD | 1.19 (1.06–1.34) | 2.07 (1.87–2.30) |
| Upper GI symptoms^d^ | 1.88 (1.72–2.04) | 2.75 (2.55–2.97) |

Abbreviations: ASA, acetylsalicylic acid; BMI, body mass index; CI, confidence interval; GERD, gastroesophageal reflux disease; GI, gastrointestinal; H_2_RA, histamine type 2 receptor antagonist; NSAID, nonsteroidal anti-inflammatory drug; OR, odds ratio; PCP, primary care physician; PPI, proton pump inhibitor; PUD, peptic ulcer disease.

^a^Relative to the indicated category, and adjusted when appropriate for sex, age, year of index date, number of PCP visits and specialist referrals in the year before the index date, smoking status, and use of gastroprotective drugs (PPIs or H_2_RAs), paracetamol, ASA and NSAIDs.

^b^None/occasional, < 3 units per week; light, 3–15 units per week; moderate, 16–24 units per week; heavy, ≥ 25 units per week).
