# Supplementary material for: Risk Factors Associated with Uncomplicated Peptic Ulcer and Changes in Medication Use after Diagnosis
Source: PLoS One. 2014 Jul 8;9(7):e101768. doi: 10.1371/journal.pone.0101768 (PMC4086954; doi:10.1371/journal.pone.0101768)
Supplement: Table S3 — Medications for which current, past or recent use was significantly associated with uncomplicated PUD development when stratified by ulcer location (gastric or duodenal). (DOC) [file pone.0101768.s003.doc]

**Table S3.** Medications for which current, past or recent use was significantly associated with uncomplicated PUD development when stratified by ulcer location (gastric or duodenal).

|  | **Uncomplicated gastric ulcer** | | **Uncomplicated duodenal ulcer** | |
| --- | --- | --- | --- | --- |
|  | **Adjusted OR^a^**  **(95% CI)** | **Crude OR^a^**  **(95% CI)** | **Adjusted OR^a^**  **(95% CI)** | **Crude OR^a^**  **(95% CI)** |
| ASA |  |  |  |  |
| Current | 1.69 (1.45–1.96) | 2.34 (2.05–2.67) | 1.36 (1.16–1.61) | 1.50 (1.30–1.74) |
| Recent | 2.13 (1.39–3.25) | 2.77 (1.87–4.12) | 1.46 (0.90–2.37) | 1.68 (1.06–2.64) |
| Past | 1.34 (0.99–1.79) | 2.09 (1.58–2.75) | 1.11 (0.80–1.54) | 1.41 (1.04–1.92) |
| NSAIDs |  |  |  |  |
| Current | 1.98 (1.68–2.33) | 3.02 (2.60–3.50) | 1.36 (1.13–1.64) | 1.55 (1.30–1.84) |
| Recent | 1.10 (0.79–1.53) | 1.72 (1.26–2.36) | 0.82 (0.56–1.21) | 1.01 (0.71–1.46) |
| Past | 1.07 (0.91–1.26) | 1.38 (1.19–1.60) | 1.00 (0.85–1.18) | 1.16 (1.00–1.34) |
| Oral anticoagulants |  |  |  |  |
| Current | 1.44 (1.01–2.06) | 1.85 (1.33–2.57) | 1.02 (0.66–1.55) | 1.09 (0.73–1.63) |
| Recent | 2.31 (1.04–5.10) | 2.02 (0.95–4.30) | 0.70 (0.23–2.16) | 0.86 (0.30–2.46) |
| Past | 2.44 (1.36–4.40) | 3.56 (2.04–6.20) | 0.99 (0.42–2.29) | 1.19 (0.53–2.70) |
| PPIs |  |  |  |  |
| Current | 1.84 (1.53–2.22) | 2.76 (2.33–3.28) | 2.03 (1.68–2.47) | 2.30 (1.92–2.75) |
| Recent | 3.52 (2.21–5.61) | 4.36 (2.81–6.75) | 4.56 (2.89–7.21) | 4.42 (2.87–6.80) |
| Past | 2.65 (2.12–3.31) | 3.65 (2.96–4.50) | 2.97 (2.37–3.73) | 3.49 (2.83–4.30) |
| H_2_RAs |  |  |  |  |
| Current | 2.77 (2.17–3.54) | 3.70 (2.93–4.66) | 3.44 (2.70–4.37) | 3.80 (3.02–4.78) |
| Recent | 3.90 (2.30–6.64) | 5.74 (3.49–9.46) | 5.43 (3.26–9.05) | 6.21 (3.80–10.13) |
| Past | 2.59 (2.05–3.28) | 3.78 (3.03–4.71) | 3.83 (3.09–4.75) | 5.08 (4.15–6.23) |

Abbreviations: ASA, acetylsalicylic acid; CI, confidence interval; H_2_RA, histamine type 2 receptor antagonist; NSAID, nonsteroidal anti-inflammatory drug; OR, odds ratio; PCP, primary care physician; PPI, proton pump inhibitor; PUD, peptic ulcer disease.

^a^Relative to non-use. Adjusted (when appropriate) according to sex, age, year of index date, number of PCP visits and specialist referrals in the year before the index date, smoking status, and use of gastroprotective drugs (PPIs or H_2_RAs), paracetamol, ASA and NSAIDs.
