# Supplementary material for: Risk Factors Associated with Uncomplicated Peptic Ulcer and Changes in Medication Use after Diagnosis
Source: PLoS One. 2014 Jul 8;9(7):e101768. doi: 10.1371/journal.pone.0101768 (PMC4086954; doi:10.1371/journal.pone.0101768)
Supplement: Table S4 — Medication use significantly associated with uncomplicated PUD development, stratified by Helicobacter pylori infection status, in a UK primary care population during 1997–2005. (DOCX) [file pone.0101768.s004.docx]

**Table S4.** Medication use significantly associated with uncomplicated PUD development, stratified by *Helicobacter pylori* infection status, in a UK primary care population during 1997–2005.

|  | ***H. pylori*-positive cases^a^** | **Controls** | **Association^b^** | ***H. pylori*-negative cases^a^** | **Controls** | **Association^b^** |
| --- | --- | --- | --- | --- | --- | --- |
|  | **n = 1,333** | **n = 9,969** |  | **n = 368** | **n = 9,969** |  |
|  | **n (%)** | **n (%)** | **OR (95% CI)** | **n (%)** | **n (%)** | **OR (95% CI)** |
| ASA | | | | | | |
| Current | 213 (16.0) | 1,133 (11.4) | 1.37 (1.14–1.65) | 57 (15.5) | 1,133 (11.4) | 1.06 (0.77–1.47) |
| Recent | 16 (1.2) | 89 (0.9) | 1.27 (0.72–2.25) | 3 (0.8) | 89 (0.9) | 0.70 (0.22–2.28) |
| Past | 45 (3.4) | 233 (2.3) | 1.24 (0.88–1.76) | 12 (3.3) | 233 (2.3) | 0.96 (0.52–1.77) |
| NSAIDs | | | | | | |
| Current | 152 (11.4) | 734 (7.4) | 1.38 (1.13–1.70) | 49 (13.3) | 734 (7.4) | 1.47 (1.05­2.07) |
| Recent | 31 (2.3) | 212 (2.1) | 0.90 (0.60–1.34) | 12 (3.3) | 212 (2.1) | 1.25 (0.67–2.32) |
| Past | 183 (13.7) | 1,303 (13.1) | 0.94 (0.79­1.13) | 71 (19.3) | 1,303 (13.1) | 1.38 (1.03–1.83) |
| Paracetamol | | | | | | |
| Current | 176 (13.2) | 868 (8.7) | 1.30 (1.07–1.59) | 56 (15.2) | 868 (8.7) | 1.41 (1.01–1.98) |
| Recent | 56 (4.2) | 281 (2.8) | 1.24 (0.90–1.71) | 20 (5.4) | 281 (2.8) | 1.63 (0.99–2.69) |
| Past | 199 (14.9) | 1,140 (11.4) | 1.15 (0.96–1.39) | 70 (19.0) | 1,140 (11.4) | 1.42 (1.05–1.91) |
| Gastroprotective medications^c^ | | | | | | |
| Current | 192 (14.4) | 733 (7.4) | 2.33 (1.94–2.80) | 78 (21.2) | 733 (7.4) | 3.11 (2.34–4.14) |
| Recent | 46 (3.5) | 83 (0.8) | 5.29 (3.62–7.74) | 15 (4.1) | 83 (0.8) | 5.36 (2.97–9.67) |
| Past | 213 (16.0) | 436 (4.4) | 4.45 (3.69–5.36) | 40 (10.9) | 436 (4.4) | 2.66 (1.86–3.82) |

ASA, acetylsalicylic acid; CI, confidence interval; H_2_RA, histamine type 2 receptor antagonist; NSAID, nonsteroidal anti-inflammatory drug; OR, odds ratio; PPI, proton pump inhibitor; PUD, peptic ulcer disease.

^a^Patients with uncomplicated PUD were classified as positive or negative for *H. pylori* or status unknown; only data on patients with known *H. pylori* status are presented.

^b^Relative to non-use. Odds ratios were calculated by comparing *H. pylori-*positive cases or *H. pylori-*negative cases with the overall control population and adjusted (when appropriate) for sex, age, year of index date, number of PCP visits and specialist referrals in the year before the index date, smoking status, and use of gastroprotective drugs (PPIs or H_2_RAs), paracetamol, ASA and NSAIDs.

^c^PPIs and/or H_2_RAs.
