# Supplementary material for: Risk Factors Associated with Uncomplicated Peptic Ulcer and Changes in Medication Use after Diagnosis
Source: PLoS One. 2014 Jul 8;9(7):e101768. doi: 10.1371/journal.pone.0101768 (PMC4086954; doi:10.1371/journal.pone.0101768)
Supplement: Table S5 — Association between PPI use and uncomplicated PUD development in naïve current ASA users. (DOC) [file pone.0101768.s005.doc]

**Table S5.** Association between PPI use and uncomplicated PUD development in naïve current ASA users.

|  | **Adjusted OR^a^**  **(95% CI)** | **Crude OR^a^**  **(95% CI)** |
| --- | --- | --- |
| No PPI | 1.00 | 1 |
| PPI at first ASA prescription | 1.27 (0.79–2.04) | 1.39 (0.88–2.17) |
| Continuous until index date | 0.86 (0.42–1.78) | 0.97 (0.48–1.93) |
| Non-continuous | 1.66 (0.91–3.04) | 1.79 (1.00–3.18) |
| PPI added after first ASA prescription | 2.29 (1.45–3.63) | 2.64 (1.71–4.05) |

Abbreviations: ASA, acetylsalicylic acid; CI, confidence interval; NSAID, nonsteroidal anti-inflammatory drug; OR, odds ratio; PCP, primary care physician; PPI, proton pump inhibitor; PUD, peptic ulcer disease.

^a^Relative to non-use of a PPI, and adjusted (when appropriate) according to sex, age, year of index date, number of PCP visits and specialist referrals in the year before the index date, smoking status, and use of paracetamol and NSAIDs.
